# Insights into the Safety and Versatility of 4D Printed Intravesical Drug Delivery Systems

**DOI:** 10.3390/pharmaceutics15030757

**Published:** 2023-02-24

**Authors:** Marco Uboldi, Cristiana Perrotta, Claudia Moscheni, Silvia Zecchini, Alessandra Napoli, Chiara Castiglioni, Andrea Gazzaniga, Alice Melocchi, Lucia Zema

**Affiliations:** 1 Sezione di Tecnologia e Legislazione Farmaceutiche “Maria Edvige Sangalli”, Dipartimento di Scienze Farmaceutiche, Università degli Studi di Milano, via Giuseppe Colombo 71, 20133 Milano, Italy; 2 Dipartimento di Scienze Biomediche e Cliniche, Università degli Studi di Milano, via Giovanni Battista Grassi 74, 20157 Milano, Italy; 3 Dipartimento di Chimica, Materiali e Ingegneria Chimica “Giulio Natta”, Politecnico di Milano, piazza Leonardo da Vinci 32, 20133 Milan, Italy

**Keywords:** 3D printing, cytotoxicity, controlled release, fused deposition modeling, local delivery, retentive systems, shape memory polymers

## Abstract

This paper focuses on recent advancements in the development of 4D printed drug delivery systems (DDSs) for the intravesical administration of drugs. By coupling the effectiveness of local treatments with major compliance and long-lasting performance, they would represent a promising innovation for the current treatment of bladder pathologies. Being based on a shape-memory pharmaceutical-grade polyvinyl alcohol (PVA), these DDSs are manufactured in a bulky shape, can be programmed to take on a collapsed one suitable for insertion into a catheter and re-expand inside the target organ, following exposure to biological fluids at body temperature, while releasing their content. The biocompatibility of prototypes made of PVAs of different molecular weight, either uncoated or coated with Eudragit^®^-based formulations, was assessed by excluding relevant in vitro toxicity and inflammatory response using bladder cancer and human monocytic cell lines. Moreover, the feasibility of a novel configuration was preliminarily investigated, targeting the development of prototypes provided with inner reservoirs to be filled with different drug-containing formulations. Samples entailing two cavities, filled during the printing process, were successfully fabricated and showed, in simulated urine at body temperature, potential for controlled release, while maintaining the ability to recover about 70% of their original shape within 3 min.

## 1. Introduction

Over the years, various strategies have been investigated to improve the local treatment of bladder diseases, having as the ultimate target the achievement and maintenance of effective levels of drugs at the target site [1,2,3,4,5]. In this respect, avoiding repeated catheterizations, which are responsible for a dramatic decrease in patient compliance towards intravesical administration, and improving adherence as well as penetration of the administered drug into the urothelium still represent the main challenges to be overcome. Liquid formulations able to undergo an increase in their viscosity once at the target site, through the formation of gels at body temperature, were recently proven able to ensure long-lasting residence in the bladder coupled with controlled release [6,7,8]. Drug delivery systems (DDSs) either capable of floating into the urine or of avoiding early elimination from the target site during physiological urination thanks to a swift expansion were also proposed [9,10,11] They were generally designed to be administered via catheter and, once exhausted, to be removed in the same way or to be spontaneously eliminated following solubilization, erosion and rupture phenomena. In more detail, the so-called expandable systems could be retained in the desired organ either following an increase in their size or a controlled variation in the relevant geometry. Notably, the resulting DDSs should not damage the bladder walls or interfere with their physiological contraction.

Expandable retentive systems can be classified based on the process driving the relevant increase in spatial encumbrance, which may rely on the removal of an external constraint of a mechanical nature (e.g., exit of the system from the catheter) or on the shape memory effect (SME) provided by so-called smart materials [12,13]. The latter mechanism consists in the recovery of an original shape obtained under manufacturing, triggered by an external stimulus of a non-mechanical nature, such as a change in temperature, moisture or light [14,15,16,17]. By way of example, the first category entails the LiRIS system, for the controlled release of lidocaine, and a S-shaped device, manufactured via stereolithography 3D printing and proposed by Xu and colleagues [9,18,19,20,21].

As far as applications relying on SME are concerned, various systems have been described over years, especially for biomedical applications (e.g., scaffolds, hemostatic plugs and devices for cellular surgery) [22,23,24,25]. In this respect, the advent of 3D printing has further prompted research into shape memory polymers (SMPs) [26,27,28,29]. Indeed, during this process, the final item is manufactured, layer-by-layer, reproducing a shape previously designed through computer-aided design software [30,31]. As a consequence, besides offering high flexibility and geometric freedom, it would allow the modification, in real-time, of the product in order to fulfill specific needs, all features that would be particularly interesting for R&D and customization purposes. Among the 3D printing techniques available, fused deposition modeling (FDM) has emerged as one of the most studied in pharmaceutics, probably in view of the limited cost of the equipment and its ease of use [32,33,34]. During FDM, polymer wires, generally known as filaments and manufactured by hot melt extrusion (HME), are fed into the printhead. Here, the filament is heated and extruded through a nozzle on a build plate. The reciprocating movement of the printhead and of the build plate ensures the deposition of the molten material layer-by-layer until the product is completed from the bottom up.

Focusing on the use of SMPs for the development of organ-retentive systems by FDM, the programmed shape-shifting of a pharmaceutical-grade poly(vinyl alcohol) (PVA) was recently leveraged to develop drug-embedded matrix-type DDSs for prolonged maintenance and release into hollow muscular organs, including the bladder and the stomach [35,36]. Although water-induced SME of PVA was already described in the material-related literature, especially upon chemical modification of the polymer or relevant blending with other compounds, in this case the shape changes were demonstrated to mainly depend on contact with body temperature [37,38,39,40,41]. In more detail, samples having different original shapes, endowed with such spatial encumbrance as to avoid rapid emptying through the sphincters of the selected organs, were produced by HME and FDM. In this respect, modifications occurring on a 3D material configuration over time, triggered by an external stimulus of a non-mechanical nature and resulting in macroscopic shape changes, has been associated with the concept of 4D printing, in which time represented the fourth dimension [15,42,43,44]. Indeed, the resulting PVA-based prototypes turned out able to take on, after production, a temporary collapsed shape and to quickly recover the original one in the desired environment. As the temporary shape would ease administration, it has been conceived according to the particular features of the route selected for reaching the target organ. The possibility of using film-coating to improve mechanical resistance and timescale of release provided by the matrix-like specimens, without impairing their working mechanism, was also demonstrated [45,46].

In the present work, a further step in the development of expandable bladder-retentive DDSs based on the smart behavior of PVA was undertaken. This was done to enable novel therapeutic approaches towards urothelial bladder cancer and many other disabling pathological conditions affecting this organ (e.g., interstitial cystitis, infections), thus reducing dropouts and providing patients with more personalized, effective and tolerated treatments. In more detail, a preliminary biocompatibility study involving the evaluation of cytotoxicity on bladder cancer and human monocytic cell lines was carried out on the materials employed so far for the manufacturing of uncoated and coated PVA-based prototypes. Moreover, taking advantage of the rapid prototyping capabilities of FDM, the feasibility of an improved design for an intravesical delivery system was considered, entailing 4D printed specimens provided with internal cavities. This evolution would make it possible to overcome the limitations related to the thermal stability of drugs embedded in the PVA-based material, which needs to be processed at temperatures ≥ 180 °C, while enhancing the versatility of the DDS proposed in terms of formulations to be conveyed and achievable release performance. Indeed, the reservoir units could not only be employed for the administration of separate doses of active molecules that are mutually incompatible, but also filled with new formulations, for instance, graphene-based nanoparticles already under development [47].

## 2. Materials and Methods

### 2.1. Materials

Prototype manufacturing and physio-technological characterization: PVA05 and PVA48 (Gohsenol™ EG 05P and 48P, Mitsubishi Chemical, Tokio, Japan); glycerol (Pharmagel, Milan, Italy; GLY); methacrylic acid copolymers, Eudragit^®^ RS 100 and RL 100 (Evonik, Essen, Germany); ready-to-use dispersion of methacrylic acid copolymers, Eudragit^®^ NE (Evonik, Essen, Germany); triethyl citrate (TEC; Sigma Aldrich, Darmstadt, Germany); ethanol (Sigma Aldrich, Darmstadt, Germany); acetaminophen for direct compression (Rhodia, Milan, Italy; AAP); PLA filament (TreeDfilaments, Milan, Italy); simulated urine fluids (NaCl 13.75 g/L; MgSO_4_ 1.69 g/L; MgCl_2_ 0.83 g/L; CaCl_2_ 0.67 g/L, KCl 0.38 g/L and urea 17.40 g/L; pH 7.50).

In vitro studies: L-Glutamine, penicillin-streptomycin, Trypsin-EDTA, RPMI1940, Dulbecco’s phosphate saline buffer (PBS) w/o calcium and magnesium and fetal bovine serum (FBS) (Euroclone, Milan, Italy). Minimum Essential Medium Eagle (EMEM), 3-[4,5-dimethylthiazol-2-yl]-2,5-diphenyl tetrazolium bromide (MTT), dimethylsulfoxide (DMSO), Phorbol 12-myristate 13-acetate (PMA), lipopolysaccharides (LPS) from Escherichia coli, paraformaldehyde (PFA), TritonX-100, Fluoroshield mounting medium, Epirubicin and Mitomycin C (Sigma-Aldrich, Darmstadt, Germany). Trypan blue stain, iScript gDNA clear cDNA synthesis kit, PureZOL RNA isolation reagent, 4′,6-diamidine-2′-phenylindole dihydrochloride (DAPI) nuclear staining dye and Universal SYBR Green supermix (Bio-Rad, Hercules, CA, USA). Ki67 antibody (Abcam, Cambridge, UK). Goat serum (GS), goat anti-rabbit IgG (H + L) cross-adsorbed secondary antibody Alexa Fluor 647, MitoTracker™ Orange and Fluorescein phalloidin (ThermoFisher, Milan, Italy).

### 2.2. Methods

#### 2.2.1. Preparation of PVA-Based Formulations

PVA05 and PVA48 powders were kept in an oven at 40 °C for 24 h prior to use. Relevant formulations containing 15% by weight of GLY (calculated on the dry polymer) were prepared by kneading. Either PVA05 or PVA48 was placed in a mortar, and the liquid plasticizer was added dropwise under continuous mixing. The resulting mixtures were oven-dried at 40 °C for 8 h. Afterwards, aggregates were ground by means of a blade mill, and the <250 µm powder fraction was recovered.

#### 2.2.2. HME

HME was carried out taking advantage of a twin-screw extruder (Haake™ MiniLab II, Thermo Scientific, Milwaukee, WI, USA) equipped with counter-rotating screws and a custom-made aluminum circular die (ø = 1.80 mm), as previously described [48]. The extrusion temperature and screw speed were set at 180 °C and at 100 rpm, respectively, while the maximum torque registered was approximately 150 N·cm. Extruded rods were cut into 50 mm-long samples that were employed, as such or after coating, for in vitro toxicity studies. PVA05-based rods were also employed to feed the FDM printer. In this case, they were manually pulled and forced to pass through a caliper set at 1.80 mm and connected to the extruder. This was done to counteract possible swelling phenomena and to calibrate the rod diameter, thus enhancing the yield of filaments suitable for 3D printing (i.e., 1.75 ± 0.05 mm). After cooling, the filament diameter was verified every 5 cm in length, and portions out of specifications were discarded. Indeed, filaments with a diameter greater than 1.80 mm were unsuitable for printing.

#### 2.2.3. 3D Printing

I-shaped prototypes were fabricated using a Kloner3D 240^®^ Twin printer (Kloner3D, Florence, Italy) using computer-aided design (CAD) files purposely developed, as described in the Results and Discussion section. These were designed using Autodesk^®^ Autocad^®^ 2016 software version 14.0 (Autodesk Inc., San Francisco, CA, USA), saved in .stl format and imported to the 3D printer software (Simplify 3D, Milan, Italy). The printing parameters set for printing the PVA-based formulation are reported in Table 1.

The printing process was interrupted at a specific height (i.e., 25th layer) to enable manual filling of the system cavities with a previously weighted (≈20 mg each cavity; analytical balance, Gibertini, Milan, Italy) amount of free-flowing AAP powder, selected as the drug tracer.

**Figure 1:**
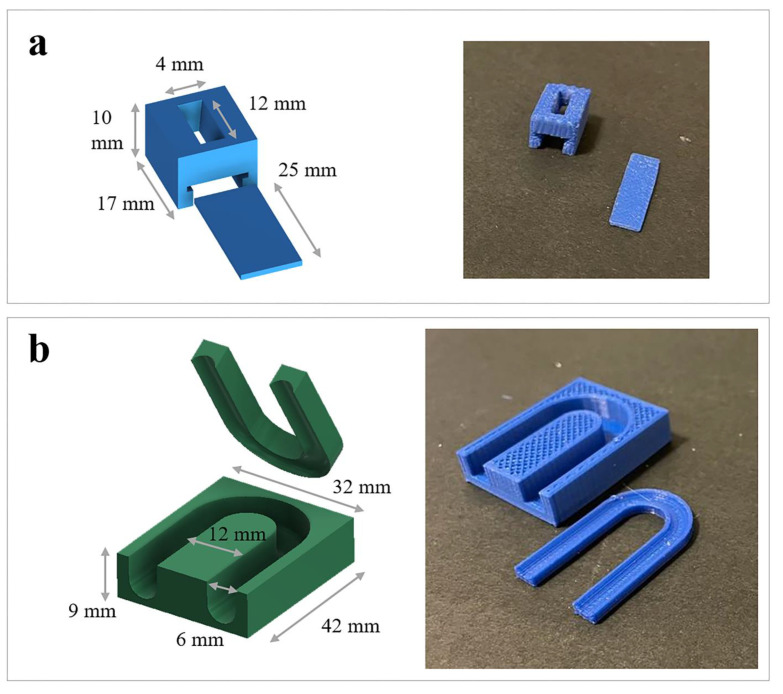
Digital models with dimensional details of (**a**) the trapdoor tool and (**b**) the template used for programming the temporary shape, together with photographs of the actual printed objects.

Using a commercial PLA filament as received, FDM was also employed to fabricate (i) a trapdoor tool to improve manual filling of the system cavities during the relevant fabrication and (ii) templates intended to make programming of samples in the desired temporary U shape easier and more reproducible (Figure 1). The printing parameters set for printing the PLA filament are reported in Table 2.

#### 2.2.4. Film-Coating

Extruded rods and printed I-shaped prototypes were coated with (i) an ethanolic solution (final concentration 30% weight/volume) containing Eudragit^®^ RS and RL (mixed in a 3:1 ratio by weight) and TEC as the plasticizer (15% by weight on the dry polymeric blend) and (ii) a 30% ready-to-use aqueous suspension of Eudragit^®^ NE. While the former samples were referred as Eudragit^®^ RS/RL-coated, the latter were identified as Eudragit^®^ NE-coated. For simplicity reasons, within the Figures, they were identified as either RS/RL or NE.

Film-coating was performed by means of an in-house assembled machinery previously described, but adapting the procedure [45]. In this respect: (i) samples were inserted in the mandrels of the equipment for half of their length; (ii) the orientation of the spray gun was modified to assume an angle of 120° with respect to the horizontal plane, cutting the cylindrical samples on their major axis, thus enabling coating of the lateral surface and of the free end of the sample at the same time; (iii) the coating process was carried out for overall 4 min, being paused after 2 min to allow extraction of the specimen from the mandrel and its 180° rotation. This way, the prototypes were flipped, thus enabling coating of the half of the sample that was previously fixed in the mandrel. At the end of the process, coated specimens were maintained for 2 h in a ventilated oven set at 40 °C.

#### 2.2.5. Physio-Technological Characterization

**Figure 2:**
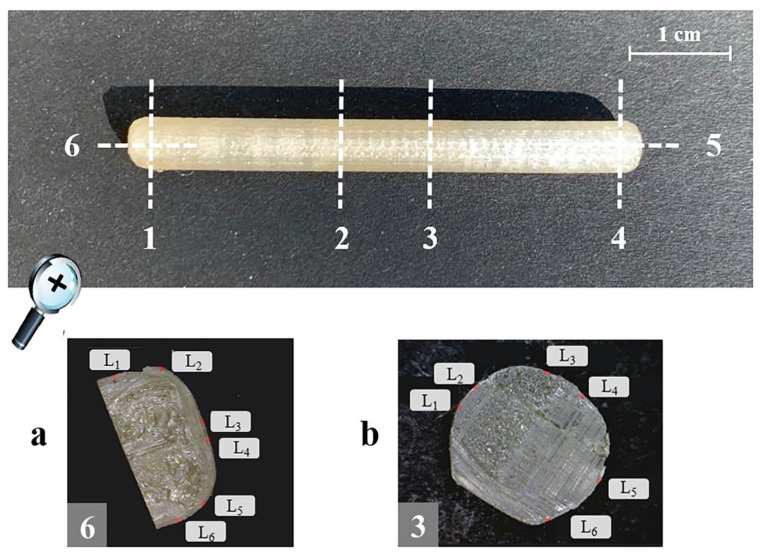
Outline of the positions in which each I-shaped sample was cut, together with photographs of the resulting cross-sections (types a and b). By way of example, details relevant to the thickness measurements (L_1_–L_6_) of the coating layer taken at position 3 and 6 are highlighted.

All the specimens were characterized for weight (*n* = 6; analytical balance, Gibertini, Milan, Italy). The thickness of the coating layer was also evaluated (*n* = 6). For this purpose, each sample was cut in six positions, i.e., one to four along its length and five and six on the ends (Figure 2). Notably, the positions in which the specimens were cut were selected to avoid the areas of the printed samples intended for drug filling. Photographs of each cross-section were acquired using a digital microscope (Digital Microscope AM-413T, Dino-Lite, Milan, Italy; resolution = 1.3 megapixel − 1280 × 1024) and processed by a dedicated software (ImageJ, Milan, Italy) to measure the thickness of the coating at six different points (L_1_–L_6_) along the circumference of each cut surface.

The SME was evaluated as previously described [35] using a purposely developed shape memory cycle, first involving the programming of the temporary shape and then recovery of the original one. The programming phase was carried out by heating the I-shaped samples up to 55 °C (i.e., at least 20 °C above their T_g_) (oven, VWR, Milan, Italy). By means of the purposely printed template (see Figure 1b), which was also stored at 55 °C, the specimens were programmed to take on the temporary U shape. This step was manually performed. In more detail, the prototype was bent and positioned at the bottom part of the template (i.e., that resembling a U-shaped cavity), which was then closed by the relevant cover. Finally, the entire assembly maintaining the sample in the desired temporary configuration was cooled at −20° C for at least 8 h (Freezer, VWR, Milan, Italy). Recovery of the original shape was triggered upon immersion of the deformed specimens (*n* = 3) into 100 mL of unstirred simulated urine fluid, prepared as reported by Sherif and colleagues [49]. The latter was kept at 37 ± 0.5 °C, using a thermoregulated bath. The recovery process was monitored using a digital camera positioned at 10 cm above the samples (GoPro Hero Session, San Mateo, CA, USA). The photographs collected were processed by means of a specific software (ImageJ, Milan, Italy) to measure the variation of the angle between the two arms (α) of the samples so as to quantify the recovery of the original shape over time. Indeed, a recovery index (RI) versus time curves were then built, with RI calculated as follows:

(1) $$\mathrm{RI}=\;\frac{\alpha -\alpha_{p}}{\pi -\alpha_{p}}$$

where α_p_ is the angle obtained in the programming phase (angles in rad).

Uncoated and coated 3D printed prototypes, the inner cavities of which were filled with AAP during relevant fabrication, were tested for release by means of a USP38 dissolution apparatus 2 (10 rpm, 37 ± 0.5 °C; Distek, North Brunswick Township, NJ, USA; *n* = 3). A total of 400 mL of the abovementioned simulated urine fluids were used as the dissolution medium. The apparatus was connected to a pump (IPC Ismatec™, Thermo Fisher Scientific, Milan, Italy) for automatic collection of fluid samples and to a spectrophotometer for relevant assay (Lambda 35, Perkin Elmer, Milan, Italy; 1 mm cuvette path length, 248 nm λ_max_). In this respect, AAP was selected as the drug tracer in light of its safety of use and based on the availability of a routine spectroscopic assay already developed. The amount of drug released at each time point was determined from a dedicated calibration curve (y = 6.43072x, R^2^ = 0.9999; from 0.0125 to 0.40 mg/mL). Besides selecting the suitable range of drug concentrations to be tested during the initial set-up phase, the presence of excipients (i.e., PVA and GLY) in the dissolution medium was demonstrated not to affect the spectroscopic AAP determination. By linear interpolation of the release data immediately before and after the time point of interest, times to 10% and 90% release (i.e., t_10%_ and t_90%_, respectively) were calculated. While t_10%_ defined the lag phase, t_90%_ was used to calculate the pulse time (i.e., t_90%_–t_10%_).

#### 2.2.6. In vitro Toxicity Studies

##### Cell Culture

In vitro studies were performed using the human bladder cancer HT1376 and the human monocytic THP-1 cell lines. HT1376 cells were obtained by American Type Culture Collection (ATCC), while THP-1 cells were kindly provided by Dr. Irma Saulle, Department of Pathophysiology and Transplantation, Università degli Studi di Milano. HT1376 cells were routinely cultured as a monolayer in Minimum Essential Medium Eagle supplemented with 10% heat inactivated FBS, 1% penicillin/streptomycin and 1% L-Glutamine. THP-1 cells were maintained in suspension in RPMI1640 supplemented with 1% L-glutamine, 1% streptomycin/penicillin and 20% FBS. For differentiation into macrophages, cells were seeded in 6-well plates at a confluence of 6 × 10^5^ cells/well and treated for 24 h incubation with 50 ng/mL phorbol 12-myristate 13-acetate (PMA) [50,51]. For polarization toward a proinflammatory phenotype, macrophages were incubated with 250 ng/mL of LPS for 48 h.

##### Cell Viability, Proliferation and Death

Viability of HT1376 cells was assessed by MTT assay [52,53,54]. Cells were seeded into 6-well plates (2 × 10^5^ cells per well) and incubated for 24 h. Then, the specimens (4 mm in length) were placed in direct contact with the cells or onto a transwell insert (0.4 μm pores) into the culture medium. Cells cultured in the medium without adding the specimens were taken as the negative control, while cells cultured in the presence of a solution (1 μM) of the chemotherapeutic drug epirubicin were used as the positive control. After 24–48 h of incubation, a MTT dye working solution was added to each well (final concentration 0.5 mg/mL). After 3 h of incubation, the supernatant was removed and replaced by 100 μL/well of DMSO. The absorbance (A) values of each well were recorded at 560 nm on an automatic plate reader (Glomax, Multi Detection System microplate reader, Promega, Milan, Italy). The relative viability versus the untreated control cells was calculated as follows:

(2) $$\mathrm{Relative}\;\mathrm{viability}\;\left(\%\right)=\frac{A_{\mathrm{exposed}\;\mathrm{group}}}{A_{\mathrm{control}}}\times 100$$

Cell proliferation and death were assessed by immunofluorescence [55,56,57]. Cells treated as previously described were incubated with staining solution containing MitoTracker^®^ fluorescent probe for 45 min in the dark, to analyze cell death. Then, cells were fixed in 4% PFA in 0.1 M PBS (pH 7.4) for 15 min, permeabilized with 0.1% TritonX-100 in PBS for 5 min and incubated in blocking buffer containing 5% normal goat serum and 0.1% TritonX-100 in PBS for 1 h. The primary antibody against Ki67, a proliferation marker, and Alexa Fluor conjugated secondary antibody were diluted in blocking buffer and incubated at 4 °C overnight and for 2 h at room temperature, respectively. Fluorescein phalloidin was used for cytoskeleton (actin) detection and incubated together with the secondary antibody. Nuclei were counterstained with DAPI Nuclear Staining Dye for 10 min at room temperature. Confocal imaging was performed with a Leica TCS SP8 AOBS microscope system with oil immersion objective 40X/1.30 (Leica, Heerbrugg, Switzerland). Image acquisitions were controlled by the Leica LAS AF software (Leica, Heerbrugg, Switzerland). Image analysis was performed with the ImageJ software (ImageJ, Milan, Italy).

##### Cytokine Analysis by Real-Time PCR

The analysis of the mRNA expression of cytokines was performed as previously described [54,57]. Total RNA from THP-1 derived macrophages was extracted with the PureZol RNA Isolation Reagent (Bio-Rad, Hercules, CA, USA), according to the manufacturer’s protocol. First-strand cDNA was generated from 1 µg of total RNA using iScript Reverse Transcription Supermix (Bio-Rad, Hercules, CA, USA). A set of primer pairs (Eurofins Genomics, Milan, Italy) was designed to hybridize to unique regions of the appropriate gene sequence (Table 3). PCR was performed using SsoAdvanced Universal SYBR Green Supermix and the CFX96 Touch Real-Time PCR Detection System (Bio-Rad, Hercules, CA, USA). The fold change was determined relative to the control after normalizing to GAPDH and Rpl32 (internal standard) through the use of the formula 2^−ΔΔCT^.

##### Statistical Analysis

Statistical significance of raw data between the groups was evaluated using one-way ANOVA followed by Tukey post-tests (multiple comparisons). The analysis was carried out by using GraphPad Prism software package (GraphPad Software, San Diego, CA, USA). The results are expressed as means ± SEM of the indicated *n* values. *p* values ≤ 0.05 were considered statistically significant.

## 3. Results and Discussion

### 3.1. Cytotoxic Evaluation of PVA-Based Samples

**Figure 3:**
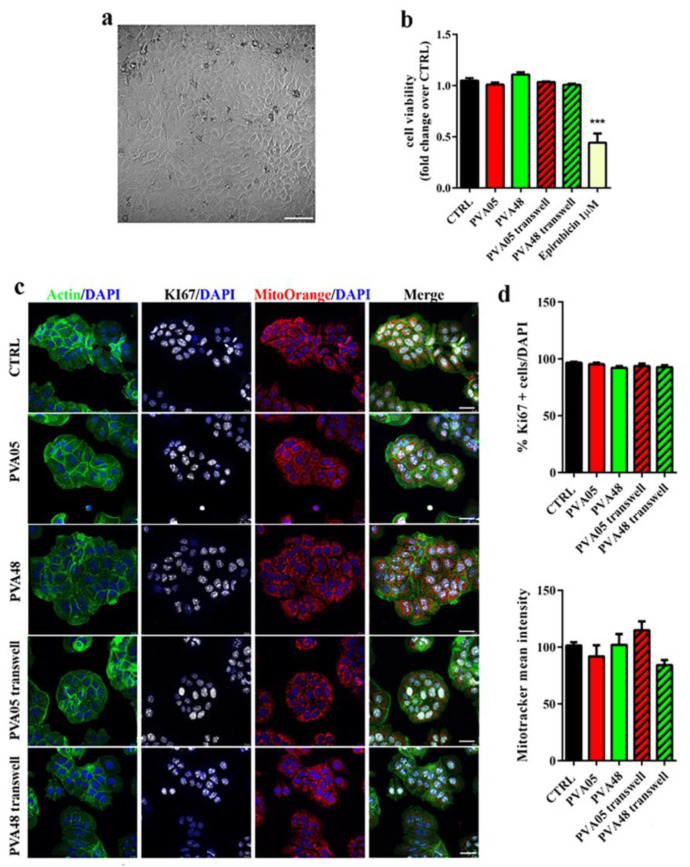
Cytotoxicity evaluation of uncoated PVA05- and PV48-based specimens. (**a**) Brightfield microscopy image of HT1376 cells (100 µm scale bar); (**b**) cell viability of HT1376 cells exposed to different samples for 24 h (*n* = 6). Untreated cells (CTRL) and epiribicin-treated cells were used as negative and positive controls, respectively (*** *p* < 0.0001 versus CTRL); (**c**) confocal microscope images of HT1376 cells stained with phalloidin to detect actin (green), Ki67 (white), Mitotracker Orange (red) and DAPI (blue) (20 µm scale bar); (**d**) upper panel: graph of the % of Ki67+ cells over total cells counted by DAPI staining; bottom panel: graph of the mean fluorescent intensity of Mitotracker Orange (*n* = 3).

In order to preliminarily evaluate the safety impact of the expandable bladder-retentive DDS under development, which involves pharmacopeial-grade materials of established safety by oral intake but processed through new hot melt technologies, a cytotoxicity study was carried out according to a predefined protocol. HME prototypes, based on two different PVA grades, uncoated and coated with Eudragit^®^ RS/RL and NE formulations, were considered. The analyses were carried out in a cancer cell line of bladder origin (i.e., HT1376). The reason for the choice of this type of cells was twofold: (i) they are a good model of bladder carcinoma, widely used to evaluate efficacy of anticancer treatments [58]; (ii) they still maintain epithelial features (Figure 3a) and therefore may be considered a valuable model for any epithelium [59]. According to ISO 10993-Biological evaluation of medical devices Part 5: Tests for in vitro cytotoxicity, the prototypes were incubated either in direct physical contact with cultured cells or placed onto a transwell insert into the culture medium to allow an indirect contact with the cells. Cytotoxicity was determined by quantifying cell viability (i.e., the measure of the proportion of live, healthy cells within a population), cell proliferation (i.e., the assessment of dividing cells) and cell death (i.e., the evaluation of cells committed to death or already dead) [53]. First, cell viability upon contact with uncoated PVA prototypes was investigated by the MTT assay, using the chemotherapeutic drug epirubicin (1 μM) as positive control of toxicity [53,60]. After 24 h of incubation, none of the PVA-based specimens in either culturing condition (i.e., direct and indirect contact) caused a significant reduction of cell viability when compared with untreated control cells (Figure 3b). As expected, epirubicin showed a high toxicity by decreasing cell viability by nearly 50%. Then, cell proliferation was assessed by staining the cells for the proliferation marker Ki67, a nuclear nonhistone protein that is expressed in proliferating cells and absent in quiescent cells [61]. The percentage of Ki67+ cells in the specimen treated samples was similar to that of the control, therefore confirming no effect on cell proliferation (Figure 3c,d).

A reliable and sensitive indicator of cell stress and apoptosis (e.g., programmed cell death) is the dissipation of the mitochondrial membrane potential. For this purpose, MitoTracker^®^ Orange fluorescent and potentiometric dye, which accumulates in mitochondria within living cells but not in dying cells, was employed. No differences in the mean fluorescence intensity were observed among the samples analyzed. Moreover, no signs of apoptosis, e.g., cell and nucleus shrinkage, or condensed chromatin were highlighted [62] (Figure 3c). Taken together, these results indicated that the specimens were non-toxic to the cells, which is consistent with previous data on the good biocompatibility of PVA composites that enforced its use for different biomedical applications [63].

**Figure 4:**
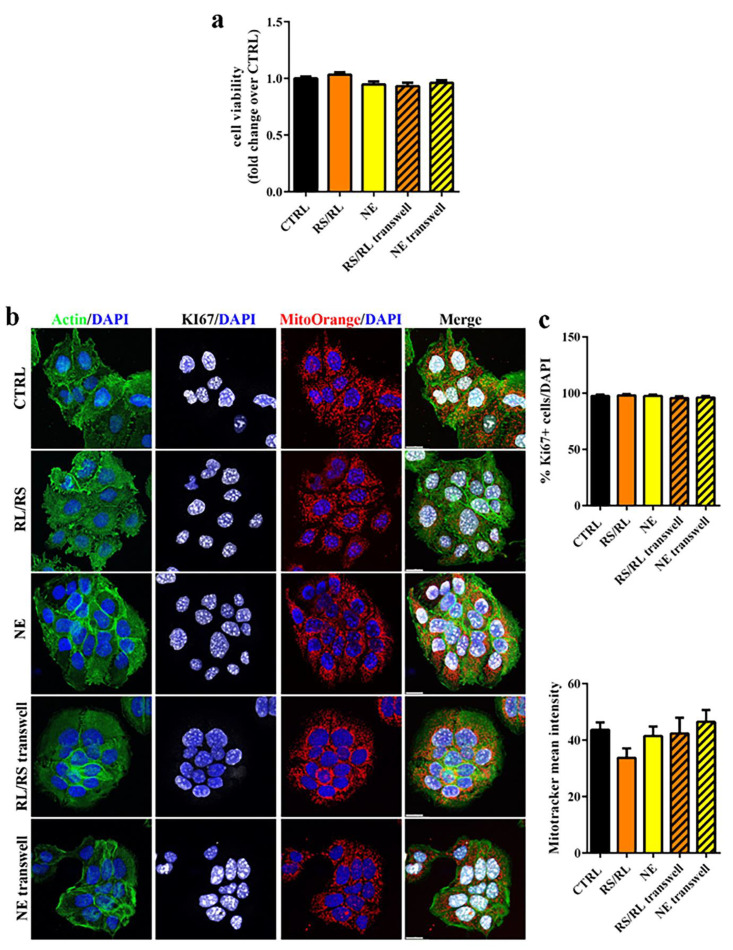
Cytotoxicity evaluation of Eudragit^®^ RS/RL- and Eudragit^®^ NE-coated specimens at 24 h of incubation. (**a**) Cell viability of HT1376 cells exposed to different samples for 24 h (*n* = 6). Untreated cells (CTRL) were used as negative control; (**b**) confocal microscope images of HT1376 cells stained with phalloidin to detect actin (green), Ki67 (white), Mitotracker Orange (red) and DAPI (blue) (20 µm scale bar); (**c**) upper panel: graph of the % of Ki67+ cells over total cells counted by DAPI staining; bottom panel: graph of the mean fluorescent intensity of Mitotracker Orange (*n* = 3).

**Figure 5:**
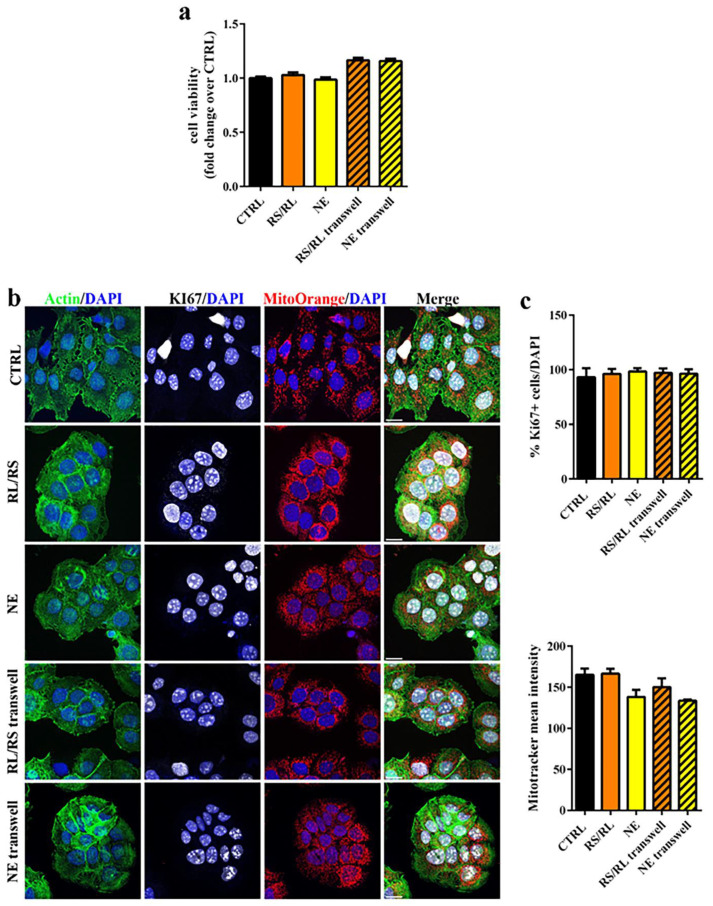
Cytotoxicity evaluation of Eudragit^®^ RS/RL- and Eudragit^®^ NE-coated specimens at 48 h of incubation. (**a**) Cell viability of HT1376 cells exposed to different samples for 48 h (*n* = 6). Untreated cells (CTRL) were used as negative; (**b**) confocal microscope images of HT1376 cells stained with phalloidin to detect actin (green), Ki67 (white), Mitotracker Orange (red) and DAPI (blue) (20 µm scale bar); (**c**) upper panel: graph of the % of Ki67+ cells over total cells counted by DAPI staining; bottom panel: graph of the mean fluorescent intensity of Mitotracker Orange (*n* = 3).

The same experimental protocol was then applied to prototypes coated with both Eudragit^®^ RS/RL and Eudragit^®^ NE formulations, which were maintained in both direct and indirect contact with the cells for 24 and 48 h (Figure 4 and Figure 5). Because no differences between PVA05 and PVA48 coated specimens were highlighted, only data relevant to the lower molecular grade polymer are reported in the following Figures. As for the uncoated specimens, no signs of toxicity, potentially caused by the presence of the prototypes, was found either after 24 or 48 h of incubation. Cells were metabolically active and healthy (Figure 4a and Figure 5a) and maintained their ability to proliferate, and no evidence of apoptosis was observed (Figure 4b,c and Figure 5b,c). This was almost expected, as Eudragits^®^ polymers are generally regarded as inactive, nontoxic and nonirritant materials [64]. Of note, this is the first report of the safety of the combination of PVA/Eudragit^®^ in a model of bladder epithelial cells. Recently, PVA-based hydrogel beads coated with Eudragit^®^ and orally administered have been tested in vivo, demonstrating the biocompatibility of the combination of such materials [65].

**Figure 6:**
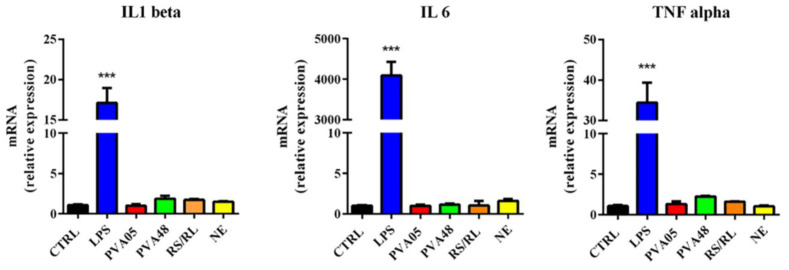
Proinflammatory cytokine expression in THP-1-derived macrophages exposed to uncoated PVA05- and PV48-based specimens and to Eudragit^®^ RS/RL- and Eudragit^®^ NE-coated ones. THP-1 cells differentiated into macrophage by the incubation with PMA for 24 h were exposed to the different samples for 48 h. Untreated macrophages (CTRL) and LPS treated macrophages were used as negative and positive controls, respectively. IL1 beta, IL6 and TNF alpha expression was analyzed by RT-PCR. Values are expressed as mean ± SEM (*n* ≥ 3) normalized versus CTRL (*** *p* < 0.001 versus CTRL).

Finally, the inflammatory potential of uncoated and coated PVA-based specimens was investigated analyzing the expression of proinflammatory cytokines (IL1beta, IL6 and TNF alpha) by monocyte-derived THP-1 macrophages [66]. Macrophages treated with LPS, capable of inducing polarization toward an inflammatory phenotype and stimulating the expression of proinflammatory cytokines, were used as positive control (Figure 6). None of the devices tested was able to modify cytokine expression compared to the untreated control, suggesting that PVA-based specimens with or without coating did not affect the macrophage inflammatory response.

Similar findings were obtained by Omata and collaborators demonstrating that PVA-based coated particles were biocompatible and nontoxic and did not induce cytokine production by macrophages [67]. On the contrary, Strehl and co-investigators noticed an increase in the production of several cytokines, comparable to an acute inflammatory process, in human macrophages stimulated with PVA-coated nanoparticles [68]. This discrepancy might be explained by the differences in phagocytosis observed for the two types of particles, the former not being internalized as opposed to the latter. Phagocytosis is indeed a critical factor in macrophage activation [69,70]. 

### 3.2. New Configuration of the PVA-Based DDS

A second objective of the work was to demonstrate the feasibility of a different configuration for the PVA-based DDS under development, entailing internal cavities for drug filling and still exhibiting the SME ensuring bladder retention. To this end, samples characterized by a rather simple I shape were selected on account of the expected ease of fabrication and programming. Indeed, they were already demonstrated to be suitable screening tools for evaluating geometric and formulation changes (e.g., application of coatings) during development of PVA-based matrix-like prototypes. Moreover, the effectiveness of their shape recovery performance turned out independent of the original/temporary shapes considered [35,36,46].

**Figure 7:**
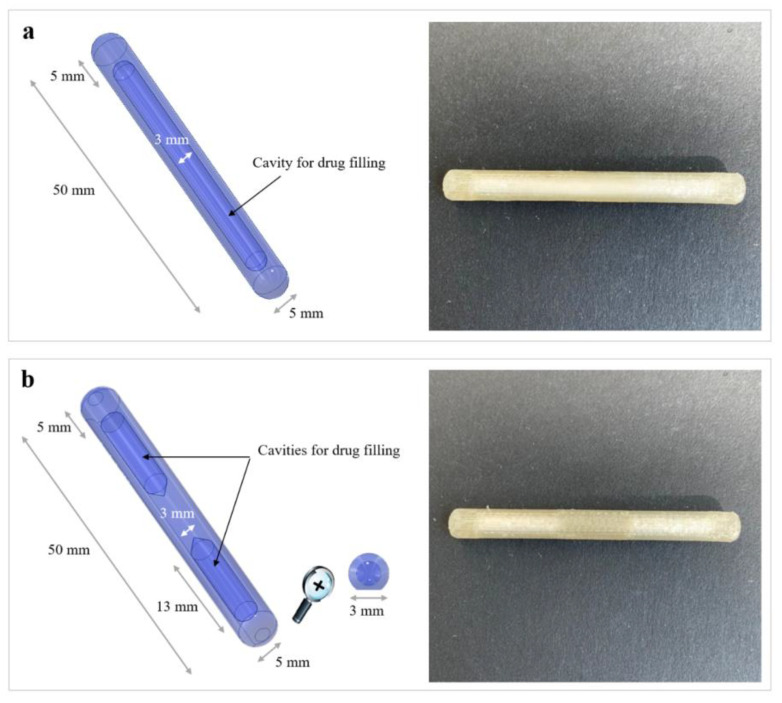
Digital models with dimensional and geometric details of (**a**) single and (**b**) two-compartment I-shaped prototypes, together with photographs of the actual printed and filled samples.

As a first attempt, an I-shaped item characterized by the presence of a single internal cavity that would occupy most of its length was conceived for FDM manufacturing (Figure 7a). While the printing of samples starting from PVA05-based filaments was successful, during the programming of the temporary U shape and recovery of the original I shape, they showed a tendency to collapse and break at the curvature. Such a behavior was independent of the wall thickness (up to 1.5 mm) considered for the samples and was associated with the limited amount of polymeric material over which mechanical stresses could be released during U bending and subsequent opening of the arms of the specimens. For this reason, a new design was proposed, entailing a solid central polymeric portion separating two independent cavities, also named as compartments (Figure 7b). The presence of two separated cavities could also improve the versatility of the delivery system, offering more filling options. Inner compartments were designed with inward-facing ends conical in shape to increase the volume of the full central portion. The pseudo-circular section (5 mm in diameter) and the rounded external edges the system was provided with were intended to allow easy positioning of the latter in a catheter considered of medium size in clinical practice (i.e., external diameter greater than 16 Ch). The cross-section of the prototypes was flattened in correspondence with the portion resting on the printing plate, thus leading to a contact surface of approximately 3 mm in width. This detail improved the adherence of the first layers during the FDM process, limiting the chances of relevant detachment and reducing the number of printing failures. Moreover, to ensure loading of drug-containing formulations into the system cavities within a single process, printing was interrupted at the 25th layer. In this respect, the first trials were carried out having the operator fill each compartment by volume and then restarting the FDM process. However, with the aim of improving the consistency obtained with the volume-dependent filling, a trapdoor tool was designed and printed (see Figure 1a). It allowed for the accurate weighting of the powder and the easy transfer of the latter into the system cavities. Indeed, the trapdoor consisted in a chamber equipped with a removable base: in the closed configuration, the trapdoor could be placed on an analytical balance and filled at will from its top opening. When positioned on top of the prototype under fabrication, the base of the chamber could be manually removed, enabling the previously weighted powder to freely flow into the cavities, still maintaining an overall process time below 15 min.

Based on the two-compartment design, prototypes with 100% and 50% infill, i.e., in principle characterized by different porosity, were printed. These proved to be quite reproducible in terms of the final weight of the device, and the variability of such parameters was reduced through the use of the semi-manual loading procedure (i.e., relying on the trapdoor tool). Based on these considerations, the weight variability observed was mainly attributed to the layer deposition mechanism typical of the 3D printing process (Table 4). Moreover, in view of the experience already gained with drug-embedded matrix-like DDSs, both 100 and 50% infill samples were coated with a low-permeable film based on Eudragit^®^ NE, which could foster changes in the release performance. Considering the new configuration devised, the coating should have been layered over the entire external surface of the I-shaped specimens, in order to avoid undesired differences in the system surface exposed to aqueous fluids and thus of rate of dissolution/erosion of the polymeric walls (i.e., reducing the risk of uncontrolled penetration of water and early opening of the cavities). In this respect, a semi-automated coating procedure was preliminarily employed using the lab-scale equipment already developed for I-shaped specimens. As expected, based on the similar external dimensions and because the mass of the specimen would not affect its ability to rotate during the coating process, weight gain and coating thickness of all the samples turned out to be reproducible and independent of the infill percentage (Table 5).

**Figure 8:**
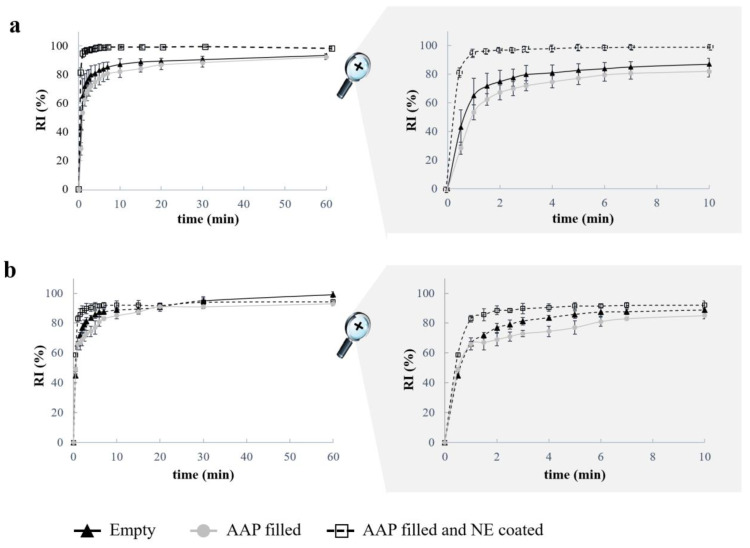
RI versus time curves relevant to different I-shaped samples (i.e., having empty reservoir units, being filled with the selected powder tracer and tested as such or after relevant coating) printed with (**a**) 100% and (**b**) 50% infill.

**Figure 9:**
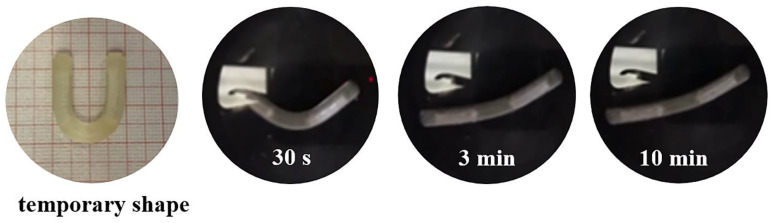
Photographs of an I sample, printed with 100% infill and filled with the drug tracer, after programming of the temporary U shape and during the recovery experiments.

When tested for shape recovery, both empty samples and those filled with the powder tracer exhibited the desired behavior regardless of their design features (i.e., infill percentage and presence of the coating) (Figure 8). In particular, neither alteration nor collapse of the compartments occurred when programming the temporary shape or during the recovery process (by way of example, see photographs in Figure 9). Moreover, when dealing with coated samples, no visible damage to the external film was highlighted. After only 3 min of contact with simulated urine at 37 °C, all prototypes were able to recover ≥70% of their original shape (Figure 8). The presence of the Eudragit^®^ NE-based coating seemed to slightly promote shape recovery by reducing the time required for its completion and increasing the relevant efficiency (i.e., higher RIs achieved sooner). The latter result was consistent with the data previously collected with matrix-like PVA-based prototypes and was associated with the flexibility of such a film, acting as a sort of rubbery envelope during shape recovery [46].

**Figure 10:**
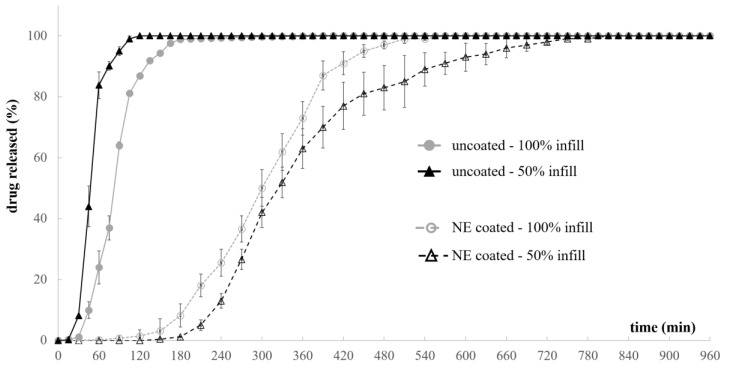
Release profiles relevant to uncoated and coated prototypes printed with different infill.

As expected, based on the new configuration and composition of the system, uncoated PVA-based prototypes pointed out a pulsatile release performance, characterized by a lag phase prior to release (i.e., t_10%_) (Figure 10) [71,72,73]. The duration of the lag phase was determined by the hydration, erosion and dissolution of the swellable/soluble PVA-based walls surrounding the drug-filled compartments, at the end of which opening of the systems occurred. Accordingly, the erosion/dissolution of the PVA walls was completed faster when these were lighter (i.e., printed setting 50% infill), resulting in lower values for both lag time and pulse time of the relevant prototypes with respect to the 100% infill samples.

Dealing with coated samples for which interaction with aqueous fluids was mediated by the presence of a poorly-permeable film, the lag time increased four- to six-fold. Indeed, the hydration and swelling of PVA occurred more slowly with respect to the uncoated samples. However, when a threshold value was reached, the volume expansion of the hydrated polymer resulted in the formation of small openings in the coating layer along the entire length of all samples. In this respect, the systems printed with 100% infill, i.e., denser and with enhanced swelling capacity, were characterized by the shortest lag phase. On the other hand, 50% infill prototypes exhibited a reduced breaking ability associated with PVA expansion, which was responsible for a reduction in the number of openings in the coating layer. The expansion was also shown to occur later, as highlighted by the greater t_10%_ value. As a consequence of the reduction in the rate of swelling and of erosion/dissolution of the PVA walls, the aqueous fluid penetrating through the openings and the swollen polymeric matrix inside the cavities might also dissolve the conveyed drug, thus promoting its diffusion outward, even before the effective opening of the reservoir cavities. This phenomenon might explain why the overall duration of release from the coated samples turned out longer with respect to the uncoated ones.

## 4. Conclusions

The availability of organ-retentive DDSs conceived to remain and release their content for a prolonged period of time into the bladder would be highly advantageous from the patient perspective, as it might reduce the number of instillations and thus of catheterizations the patient would undergo over time. While improving compliance, life-quality and relevant expectancy, this approach might also limit healthcare and social expenses by acting on administration-related costs, entailing, for instance, hospitalizations, consumables, disposal operations, commitment of hospital personnel and management of inflammations/secondary infections. In addition, retentive systems could widen the number of available treatments for bladder pathologies by implementing new therapeutic approaches combining active ingredients and involving modified time and rate of release. In this respect, the expandable intravesical DDS already proposed as a matrix-like structure for prolonged release of active molecules was here further improved to be equipped with internal cavities for extemporaneous, independent and personalized filling. The new configuration would also enable programmed release of specific drug quantities at different times.

By taking advantage of the PVA SME, this work confirmed the application potential of 4D printing in the development of DDSs intended for retention in hollow muscular organs, especially towards more complex structures (e.g., multi-layer and hollow systems). Finally, preliminary biocompatibility studies highlighted the safety of the materials used, which was particularly promising in view of the next development steps.

## Figures and Tables

**Table 1:** Operating parameters set for the PVA-based formulation.

Parameter	Value
Nozzle diameter	0.5 mm
Printing temperature	200 °C
Build plate temperature	50 °C
Extrusion flow	100% of the maximum flow
Printing speed	23 mm/s
Retraction length	2.00 mm
Retraction speed	20 mm/s
Layer height	0.10 mm
Infill	100% or 50%,
Infill geometry	Rectilinear
Number of top/bottom	2
Number of perimeters	1

**Table 2:** Operating parameters set for the PLA filament.

Parameter	Value
Nozzle diameter	0.5 mm
Printing temperature	200 °C
Build plate temperature	40 °C
Extrusion flow	100% of the maximum flow
Printing speed	65 mm/s
Retraction length	2.40 mm
Retraction speed	45 mm/s
Layer height	0.20 mm
Infill	75%
Infill geometry	Honeycomb
Number of top/bottom	3
Number of perimeters	2

**Table 3:** List of primers designed for PCR.

Gene	Primer Sequences
*IL-6*	F: 5′-GGCACTGGCAGAAAACAACC-3′ R: 5′-GCAAGTCTCCTCATTGAATCC-3′
*IL-1β*	F: 5′-TTCGACACATGGGATAACGAGG-3′ R: 5′-TTTTTGCTGTGAGTCCCGGAG-3′
*TNF*	F: 5′-CCCAGGGACCTCTCTCTAATCA-3′ R: 5′-GCTACAGGCTTGTCACTCGG-3′
*GAPDH*	F: 5′-TGAGGTCAATGAAGGGGTC-3′ R: 5′-GTGAAGGTCGGAGTCAACG 3′
*RPL32*	R: 5′-TTAAGCGTAACTGGCGGAAAC-3′ F: 5′-AAACATTGTGAGCGATCTCGG-3′

**Table 4:** Weight data relevant to uncoated I-shaped samples printed by setting different infill.

	Weight, mg (CV)	
	Manually Filled	Trapdoor-Filled
100% Infill	841.85 (9.52)	838.63 (5.02)
50% Infill	723.44 (10.11)	736.11 (5.61)

**Table 5:** Thickness and weight gain data relevant to coated I-shaped samples printed by setting different infill.

	Thickness, μm (CV)						Overall Thickness, μm (CV)	Weight Gain, % (CV)
	1	2	3	4	5	6		
100% Infill	56.43 (6.36)	55.37 (7.78)	55.75 (6.61)	51.70 (8.72)	53.27 (9.25)	55.98 (6.37)	54.75 (7.64)	6.73 (6.84)
50% Infill	54.71 (7.35)	58.03 (9.06)	52.30 (6.23)	57.05 (7.59)	54.85 (9.63)	54.54 (9.93)	55.24 (8.53)	6.22 (7.01)

## Data Availability

Data are available upon request.
